# Socioeconomic differential in self-assessment of health and happiness in 5 African countries: Finding from World Value Survey

**DOI:** 10.1371/journal.pone.0188281

**Published:** 2017-11-27

**Authors:** Oluwafunmilade Adesanya A., Bomar Mendez Rojas, Amadou Darboe, Idrissa Beogo

**Affiliations:** 1 International Health Program, National Yang Ming University, Taipei, Taiwan; 2 Ministry of Health and Social Welfare, Banjul, The Gambia; 3 School of Population and Global Health, The University of Melbourne, Melbourne, Victoria, Australia; 4 École Nationale de Santé Publique, Ouagadougou, Burkina Faso; 5 Université Laval, rue de l'Université, Québec (Québec), Canada; West Chester University of Pennsylvania, UNITED STATES

## Abstract

**Objective:**

Factors that contribute to wealth related inequalities in self-rated health (SRH) and happiness remains unclear most especially in sub-Saharan countries (SSA). This study aims to explore and compare socioeconomic differentials in SRH and happiness in five SSA countries.

**Methods:**

Using the 2010/2014 World Values Survey (WVS), we obtained a sample of 9,869 participants of age 16 and above from five SSA countries (Nigeria, Ghana, South Africa, Rwanda and Zimbabwe). Socioeconomic inequalities were quantified using the concentration index. The contribution of each predictor to concentration index’s magnitude was obtained by means of regression based decomposition analysis.

**Results:**

Poor SRH ranges from approximately 9% in Nigeria to 20% in Zimbabwe, whereas unhappiness was lower in Rwanda (9.5%) and higher in South Africa (23.3%). Concentration index was negative for both outcomes in all countries, which implies that poor SRH and unhappiness are excessively concentrated among the poorest socioeconomic strata. Although magnitudes differ across countries, however, the major contributor to wealth-related inequality in poor SRH is satisfaction with financial situation whereas for unhappiness the major contributors are level of income and satisfaction with financial situation.

**Conclusions:**

This study underscores an association between wealth related inequalities and poor SRH and unhappiness in the context of SSA. Improving equity in health, as suggested by the commission of social determinants of health may be useful in fighting against the unfair distribution of resources. Thus, knowledge about the self-rating of health and happiness can serve as proxy estimates for understanding the distribution of health care access and economic resources needed for well-being in resident countries.

## Introduction

Good health and wellbeing are critical elements of a good life and their attainment represent a major goal for individuals and nations [1]. Individuals’ self-rating of their own health and happiness has been extensively applied in population-based surveys as measures of general health and wellbeing status. Self-rated health (SRH) (or self-perceived health, or self-reported health) has been employed in sociological investigations since the 1950s [2]. A single item measure of SRH has the power to assess the general health and wellbeing of an individual putting into account the physical, psychological and social dimensions as described in the World Health Organization (WHO) definition [3–5]. A number of studies, including Epidemiological studies and meta-analysis have found strong associations between the single measure of SRH and chronic medical outcomes such as; death, diabetes, coronary heart disease, functional ability, depression, stroke, and mortality [6, 7]. Self-reported happiness (subjective well-being) on the other hand, is the subjective translation of human well-being and satisfaction with one’s life-as-a-whole [8]. It has been found to be positively correlated with SRH at both the individual and societal levels [1] and demonstrated a remarkably strong positive effect on longevity in healthy populations [8]. Hence, societies that are healthier tend to be happier and vice versa [1].

Although good health and wellbeing are critical for human life, unfortunately, there are marked inequalities in their distribution across population sub-groups and geographic locations [9]. Inequalities in health and wellbeing represent a major global health challenge. Over the past decades, attempts to unravel factors contributing to these inequalities have given rise to major theoretical development. Theories such as; fundamental causes, social selection, psychosocial theory, diffusion of innovation etc., all attempts to explain factors underpinning health inequalities within and between nations [10]. The overarching assumption of the theory of fundamental causes [11] is of relevance to findings presented in this paper. This theory stipulates that inequalities in health and well-being are mainly driven by differences in access to socio-economic opportunities (employment, education, income, power, prestige etc.) within and between countries. In this context, better socio-economic status (SES) is expected to improve health and well-being through facilitating ones’ access to fundamental resources needed for a healthy living [11, 12]. This core theoretical assumption has been supported in various population based surveys across high and low income countries (LICs) [9].

For example, bivariate analysis of cross-national data have revealed that no or less educated people are at greater risk of reporting poor health [13, 14]. Likewise, individuals from LICs are more likely to report poor health compared to those in higher income countries (HICs) [14]. A study in the United States has revealed similar findings for health and happiness [1]. The authors observed a strong income and education gradient for poor SRH and unhappiness, with the gradient being stronger for poor health [1]. Thus, health and wellbeing tent to follow a social gradient [15].

Although, several empirical analysis have consistently linked socioeconomic factors to health and wellbeing [1, 9, 16], however, the degree to which these factors contribute to inequalities in SRH and happiness remains unclear. Additionally, most of the previous self-reported investigations on health and happiness have focused on HICs [17] with more generous welfare arrangements [12]. To our knowledge, very few cross-country studies have been conducted on SRH in sub-Saharan Africa (SSA) [13, 14]. Contrary to HICs, SSA has overwhelming degree of poverty and the gap between the poor and the rich continue to widen. Fully engaged in the free market policy [18], socioeconomic-related inequalities in this region are one of the highest in the world [19]. In their investigation of SRH in Sub-Saharan Africa (SSA), Subramanian and colleagues observed an inverse association between years of schooling and poor health [1] and the strength of this association was similar for men and women.

This paper attempts to measure and compare socioeconomic differential self-assessed health and happiness across 5 SSA countries (Nigeria, Ghana, South Africa, Rwanda and Zimbabwe) using data from the world value survey (WVS) 2010–2014. These 5 countries were the only SSA countries included in the WVS 2010–2014. Notably, all 5 countries have marked income inequalities with GINI indexes above 40.00 [20]. Of the five countries, South Africa has the highest inequalities in terms of income distribution (GINI index 63.5) while Ghana has the least (42.8). SSA countries are at a crossroad of socioeconomic development and it is imperative to gauge the significant contribution of social and economic factors to health and wellbeing of the people. This paper contributes to the literature in several ways. First, the paper added to the growing body of scientific evidence on socioeconomic inequalities in SRH and happiness in the context of developing countries. Health and happiness are often discussed separately in the literature [1, 16]; hence it is imperative to examine how the two co-vary and distribute across LICs. Second, it measures socioeconomic-related inequalities in subjective health and happiness using Concentration Index (CI) of Kakawani 1980, 1997 in [21]. We employed the CI to measure and compare the degree of socioeconomic-related inequality in self-rated poor health and unhappiness. A similar approach has been used in previous studies that investigated the sources of socioeconomic-inequalities in the distribution of infant mortality [22], children malnutrition [23], poor SRH and mental health disorders [24].

## Methods

### Study design and sampling technique

We used wave 6 from the World Values Survey (WVS) 2010/2012. The survey covered 57 countries around the globe; however, we have limited our analysis to five SSA countries: Ghana, Nigeria, Rwanda, South Africa and Zimbabwe that covered by the survey. WVS is nationally representative of each country, and employs a two-stage sampling procedure to select participants of both genders aged 16 years and above. First, primary sampling units (PSUs) are randomly selected from the latest national census enumeration areas based on probability proportionate to size. PSUs commonly correspond to districts or voting stations. At a second stage, households are selected within each PSU through a systematic sampling strategy. Finally, selected respondent from each household are interviewed using face-to-face interviews. No upper age limit is imposed. The analytical sample size is composed of 9 869 respondents of both genders aged 16 to 92 years.

### Ethical approval

The dataset is available online from World Values Survey database. The WVS is approved by Institute for Comparative Survey research institutional review board.

### Outcome variable

Two outcome variables were measured namely; SRH and Happiness. In the WVS, participants’ SRH was measured using a single question with four response levels. Respondents were asked; “*all in all*, *how would you describe your state of health these days*? *With response options; very good*, *good*, *fair or poor*. We created a binary variable with 1 denoting fair or poor and 0 otherwise. Feeling of unhappiness on the other hand, was measured using the following question; “*taking all things together*, *would you say you are; very happy*, *rather happy*, *not very happy or not at all happy”*. We recoded as 1 those who answered not very happy or not at all happy and 0 otherwise. We selected unhappiness rather than happiness because the former is a proxy measure of lack of well-being i.e., unhappy individuals are more likely to be unemployed, having low education and having feelings of loneliness [25, 26].

### Predictors

We included a set of selected variables such as respondent’s gender, age, marital status, education level, satisfaction with financial situation which was categorized into 3 (completely dissatisfied, moderately satisfied, and completely satisfied). Self-reported scale/level of income was also further categorized into (lowest income, medium Income, high income, and self-reported social class (upper class, upper middle class, lower middle class, and lower class). All these predictors have been found to predict SRH and happiness [2, 3]

### Data analysis

We used descriptive analysis to assess between-country differences in the distribution of population characteristics. Next, logistic regression analyses were employed to identify predictors and estimate predicted logs for each country. No evidence of multicollinearity was found, with all variance inflation factors in each country less than 10 [27]. Then, we estimated CI [22, 28], which measures the degree that both outcomes are unequally distributed across socio-economic strata. For each outcome variable, a relative concentration curve was plotted, with the cumulative proportion of participants ranked by levels of socio-economic related variable on the x-axis and the cumulative proportion of the outcome variable on the y-axis [28]. CI is defined as twice the area between the concentration curve and the diagonal (line of equality) [29]. When the concentration curve lies above the line of equality the CI takes a negative value which indicates that the outcome of interest is concentrated among the lowest income level [28]. When the curve lies on the diagonal the value is zero (equality in the distribution of the outcome across levels of income), and when it lies below the diagonal the index takes a positive value (the outcome is more concentrated among the highest level of income). To estimate the contribution of each predictor to unequal distribution of both outcomes decomposition analysis was performed. Predicted logs obtained from logistic regression models were used at this stage. For all analyses, we used the complex survey analysis to account for a multistage sampling design and correct for unequal probabilities of selection. Bearing in mind the socioeconomic, cultural and regional diversity that characterized Africa and the necessity of providing evidence that hence the design of country-tailored interventions towards reducing socioeconomic-related inequalities in health and well-being, we decided to present the results separately by country. The analyses were conducted in Stata 12.0 and R 3.2.0.

## Results

**Table 1**depicts weighted frequencies of selected variables. Overall, there were 15% and 19% prevalence of poor health and unhappiness respectively in all the five countries. Country specific data demonstrated that proportions of self-reported unhappiness and poor health were higher among participants from South Africa and Zimbabwe. Collectively, most of the study participants (51%) were female and were under 35 years of age. Regarding socio-economic related variables, most participants (40.4%) across the five countries had at least completed secondary education. With regard to satisfaction with financial situation, most of the study participants were completely dissatisfied (47%). Self-reported household income level for participants across the five countries fall mainly within the lowest tertile (1–4). Regarding social class, most participants across the countries perceived themselves to be within the lower class.

**Table 1:** Descriptive statistics by country.

Variables	Ghana	Nigeria	Rwanda	South Africa	Zimbabwe	Total
	N = 1552	N = 1759	N = 1527	N = 3531	N = 1500	N = 9 869
	%wt^a^	%wt^a^	%wt^a^	%wt^a^	%wt^a^	%wt^a^
**Self-rated health**						
Good	87.3	90.8	82.8	83.8	80.0	84.8
Poor	12.6	9.1	17.1	16.2	19.9	15.1
**Feeling of happiness**						
Happy	80.8	84.6	90.4	76.6	78.8	81.1
Unhappy	19.2	15.3	9.5	23.3	21.1	18.8
**Gender**						
Female	49.5	48.6	50.4	51.6	54.4	51.0
Male	50.4	51.4	49.5	48.3	45.5	48.9
**Age, y**						
< 19	11.1	7.3	2.2	8.5	6.2	7.41
20 to 25	32.4	30.3	21.2	20.7	21.4	24.4
26 to 35	25.6	35.8	43.1	23.5	32.0	30.4
36 to 45	14.8	13.1	20.4	16.2	17.0	16.2
46 to 55	8.0	7.0	7.8	14.6	9.7	10.4
56 to 65	4.7	4.5	3.1	10.8	7.4	7.07
66 +	3.1	1.7	1.9	5.3	6.0	3.9
**Marital status**						
Married /cohabiting	52.0	54.4	63.8	43.6	56.6	52.0
Divorced/separated	3.8	0.4	4.1	1.9	8.5	3.3
Widowed	3.2	3.0	5.7	6.7	10.2	5.9
Single	40.9	42.0	26.2	47.6	24.5	38.8
**Education**						
No formal education/incomplete primary	26.3	16.9	22.8	7.60	9.5	14.8
Primary completed	28.2	7.9	12.0	6.1	6.7	10.9
Secondary incomplete	16.1	18.0	21.0	33.0	20.8	23.9
Secondary completed	20.9	44.5	30.7	44.3	56.4	40.4
University	8.3	12.6	13.5	8.9	6.6	9.8
**Satisfaction with financial situation**						
Completely dissatisfied (1–5)	65.8	44.3	34.8	37.5	67.8	47.3
Moderately satisfied (6–7)	19.8	30.7	43.9	31.1	21.6	29.7
Completely satisfied (8–10)	14.3	24.9	21.3	31.4	10.5	22.8
**Scales of income**						
Lowest Income (1–4)	50.81	40.08	33.01	33.72	51.00	40.10
Medium Income (5–6)	31.85	32.76	38.44	33.93	32.33	33.80
Highest Income (7–10)	17.35	27.15	28.55	29.64	16.68	25.10
Missing	0.00	0.00	0.00	2.70	0.00	0.97
**Social class**						
Upper class	2.78	2.68	1.70	1.32	3.15	2.13
Upper middle class	12.59	10.23	19.06	11.03	12.07	12.50
Lower middle class	24.68	25.41	23.18	16.54	30.02	22.40
Working class	31.90	22.61	23.97	23.79	15.58	23.60
Lower class	28.05	39.08	32.09	42.82	39.18	37.60
Don't know	0.00	0.00	0.00	4.50	0.00	1.61

%wt corresponds to weighted percentages.

**Tables 2 and 3**present results from multiple logistic regression analyses measuring socio-economic predictors of poor SRH and unhappiness across the five SSA countries. Overall, compared to those with no formal education backgrounds, participants with university degree were 26% and 23% less likely to report poor health and unhappiness, respectively. Participants who were moderately to completely satisfied with their financial situations were significantly less likely to report poor health and unhappiness relative to those who were completely dissatisfied. Additionally, compared to lowest income level group, participants with medium to high level of income were more than 30% less likely to report poor health and unhappiness. Except for South Africa, no statistically significant associations were observed between social class and health and happiness across all the other four countries.

**Table 2:** Logistic regression models linking demographic and socioeconomic characteristics with self-rated poor health.

Variables	Ghana	Nigeria	Rwanda	South Africa	Zimbabwe	Total
	N = 1552	N = 1759	N = 1527	N = 3531	N = 1500	N = 9 869
	aOR	aOR	aOR	aOR	aOR	aOR
	(95% CI)	(95% CI)	(95% CI)	(95% CI)	(95% CI)	(95% CI)
**Gender**						
Female	1	1	1	1	1	1
Male	0.66 (0.47–0.94)**	0.92 (0.64–1.34)	1.02 (0 .75–1.39)	1.09 (0 .89–1.34)	0 .98 (0 .70–1.37)	0.95 (0.84–1.08)
**Age, y**						
< 19	1	1	1	1	1	1
20 to 25	0.63 (9.34–1.17)	0.59 (0.28–1.21)	2.74 (0 .59–12.7)	1.72 (0 .88–3.35)	1.68 (0 .62–4.49)	1.16 (0.83–1.61)
26 to 35	1.06 (0.56–1.97)	1.15 (0 .57–2.32)	1.54 (0 .32–7.4)	3.07 (1.63–5.78)**	1.49 (0 .54–4.06)	1.50 (1.08–2.07)**
36 to 45	1.267 (0.61–2.59)	0 .60 (0 .24–1.47)	2.26(0 .45–11.2)	3.62 (1.88–6.97)***	2.26 (0 .80–6.37)	1.74 (1.23–2.46)**
46 to 55	1.43(0.64–3.20)	0 .82 (0 .31–2.14)	3.24 (0 .62–16.8)	5.70 (2.94–11.06)***	3.73 (1.27–10.9)**	2.60 (1.81–3.73)***
56 to 65	3.38 (1.39–8.18)**	2.04 (0 .79–5.24)	4.97 (0 .88–28.0)	10.8 (5.46–21.7)***	5.78 (1.89–17.6)**	4.75 (3.24–6.96)***
66 +	5.48 (2.11–14.2)***	1.88 (0 .51–6.91)	10.6 (1.72–66.3)**	17.07 (8.04–36.2)***	11.40 (3.43–38.0)***	8.21 (5.37–12.5)***
**Marital status**						
Married /cohabiting	1	1	1	1	1	1
Divorced/separated	1.72 (0.85–3.48)	3.87 (1.07–13.9)**	2.71 (1.45–5.06)**	0 .79 (0 .41–1.55)	1.57 (0 .93–2.65)	1.61 (1.21–2.15)**
Widowed	0 .75 (0.32–1.76)	0 .90 (0 .34–2.35)	1.47 (0 .79–2.70)	1.15 (0 .78–1.70)	1.68 (1.01–2.78)**	1.25 (0.98–1.59)
Single	1.20 (0.77–1.86)	0.90 (0.56–1.46	0 .55 (0 .33–0.91)**	1.0 (0 .77–1.29)	0 .56 (0 .34–0 .94)**	0.90 (0.76–1.06)
**Education**						
No formal education/incomplete primary	1	1	1	1	1	1
Primary completed	0.71 (0.45–1.11)	0 .94 (0 .48–1.86)	1.52 (0 .92–2.50)	1.18 (0 .74–1.89)	0 .46 (0 .23–0 .93)**	1.05 (0.84–1.32)
Secondary incomplete	0.70 (0 .39–1.22)	0 .47 (0 .24–0 .94)**	1.91 (1.18–3.12)**	0 .91 (0 .62–1.33)	0 .64 (0 .34–1.20)	1.01 (0.81–1.24)
Secondary completed	0 .65 (0 .39–1.10)	0 .74 (0 .43–1.27)	2.02 (1.26–3.22)**	0 .71 (0 .48–1.06)	0 .53 (0 .29–.98)**	0.89 (0.72–1.09)
University	0 .72 (0 .35–1.48)	0 .28 (0 .13–0 .63)**	1.70 (0 .89–3.24)	0 .60 (0 .34–1.05)	0 .70 (0 .31–1.55)	0.74 (0.55–0.99)**
**Satisfaction with financial situation**						
Completely dissatisfied (1–5)	1	1	1	1	1	1
Moderately satisfied (6–7)	0.48 (0.29–0 .77)**	0 .19 (0 .11–0 .33)***	0 .83 (0 .59–1.16)	0 .60 (0 .47–0 .78)***	0 .46 (0 .29–0 .72)**	0.56 (0.48–0.66)***
Completely satisfied (8–10)	0.42 (0.24–0.72)**	0.33 (0 .19–0 .58)***	0 .42 (0 .24–0 .73**	0 .45 (0 .34–0 .59)***	0 .44 (0 .24–0 .79)**	0.42 (0.35–0.52***
**Scales of income**						
Lowest Income (1–4)	1	1	1	1	1	1
Medium Income (5–6)	0 .84(0 .55–1.30)	0 .78 (0 .49–1.26)	0 .26 (0 .18–0 .38)***	0 .88 (0 .67–1.14)	0 .88 (0 .57–1.35)	0.68 (0.58–0.80)***
Highest Income (7–10)	0.80(0 .46–1.41)	1.02 (0.57–1.82)	0 .24 (0 .13–0 .41)***	0 .85 (0 .62–1.17)	1.01 (0 .57–1.77)	0.70 (0.57–0.85)**
**Social class**						
Upper class	1	1	1	1	1	1
Upper middle class	1.18 (0.38–3.62)	0 .85 (0 .17–4.18)	0 .65 (0 .17–2.49)	0 .82 (0 .27–2.48)	1.13 (0 .44–2.88)	0.86 (0.52–1.44)
Lower middle class	1.31 (0.43–3.96)	0.98 (0 .21–4.51)	0 .67 (0 .18–2.50)	1.20 (0 .40–3.54)	0 .82 (0 .33–2.07)	0.98 (0.59–1.60)
Working class	1.04 (0 .33–3.22)	0 .93 (0 .20–4.36)	0 .38 (0 .10–1.45)	1.30 (0 .44–3.81)	1.04 (0 .40–2.70)	0.89 (0.54–1.46)
Lower class	1.38 (0.43–4.42)	0 .81 (0 .17–3.80)	1.57 (0 .42–5.76)	2.05 (0 .70–5.98)	1.36 (0 .52–3.56)	1.47 (0.89–2.43)
**Country (ref: Nigeria)**						
Ghana						1.28 (1.0–1.64)**
Rwanda						2.31 (1.83–2.91)***
South Africa						1.64 (1.32–2.03)***
Zimbabwe						1.58 (1.25–2.01)***

** p<0.05.

***p<0.001.

**Table 3:** Logistic regression models demographic and socioeconomic characteristics with feeling of unhappiness.

**Variables**	Ghana	Nigeria	Rwanda	South Africa	Zimbabwe	Total
	N = 1552	N = 1759	N = 1527	N = 3531	N = 1500	N = 9 869
	aOR	aOR	aOR	aOR	aOR	aOR
**Gender**						
Female	1	1	1	1	1	1
Male	1.09 (0.822–1.46)	0.98 (0.74–1.30)	0.95 (0.66–1.39)	1.01 (0 .84–1.20)	1.47 (1.08–2.0)**	1.08 (0.96–1.21)
**Age, y**						
< 19	1	1	1	1	1	1
20 to 25	1.38 (0 .83–2.30)	0.60 (0.35–1.05)	0.36 (0.12–1.07)	1.47 (0 .97–2.20)	1.21 (0 .58–2.51)	1.11 (0.87–1.42)
26 to 35	1.76 (1.03–3.01)**	1.36 (0.79–2.34)	0.46 (0.15–1.44)	1.34 (0 .90–2.01)	1.54 (0 .73–3.24)	1.41 (1.10–1.80)**
36 to 45	1.64 (0.88–3.06)	1.49 (0.77–2.88)	0.57 (0.16–1.92)	1.60 (1.04–2.47)**	2.24 (1.01–4.96)**	1.58 (1.20–2.07)***
46 to 55	1.48 (0.72–3.06)	1.80 (0.87–3.70)	0.62 (0.17–2.29)	1.63(1.04–2.57)**	1.54 (0 .63–3.74)	1.56 (1.16–2.09)**
56 to 65	1.43 (0.57–3.55)	2.55 (1.17–5.58)**	1.02 (0.25–4.12)	1.36 (0 .81–2.29)	2.99 (1.17–7.67)**	1.69 (1.21–2.36)**
66 +	0.85 (0.25–2.86)	1.40 (0.43–4.61)	1.21 (0.27–5.44)	1.64 (0 .88–3.04)	1.96 (0 .68–5.62)	1.56 (1.04–2.33)**
**Marital status**						
Married /cohabiting	1	1	1	1	1	1
Divorced/separated	0.92 (0.451–1.87)	0.99 (0.25–3.83)	3.01 (1.59–6.05)**	0 .72 (0 .38–1.38)	1.35 (0 .80–2.26)	1.18 (0.88–1.59)
Widowed	0.54 (0.21–1.41)	1.14 (0.51–2.54)	3.22 (1.70–6.14)***	0 .96 (0 .63–1.47)	1.91 (1.13–3.23)**	1.30 (1.01–1.68)**
Single	0 .96 (0.68–1.36)	1.10 (0.76–1.59)	1.10 (0.59–2.04)	1.01 (0 .81–1.26)	0 .96 (0 .63–1.46)	1.10 (0.95–1.27)
**Education**						
No formal education/incomplete primary	1	1	1	1	1	1
Primary completed	0.90 (0.61–1.33)	1.21 (0.670–2.18)	2.55 (1.45–4.51)**	0 .91 (0 .56–1.45)	0 .57 (0 .27–1.21)	1.13 (0.90–1.42)
Secondary incomplete	1.40 (0.66–1.65)	1.31 (0.67–1.94)	2.17 (1.22–3.90)**	1.06 (0 .74–1.53)	0 .96 (0 .50–1.83)	1.26 (1.03–1.54)**
Secondary completed	0.73 (0.47–1.14)	1.55 (0.98–2.45)	1.46 (0.81–2.65)	0 .86 (0 .59–1.26)	1.17 (0 .63–2.20)	1.12 (0.91–1.36)
University	0.57 (0.31–1.06)	0.73 (0.41–1.32)	1.38 (0.62–3.09)	0 .78 (0 .46–1.32)	0 .90 (0 .39–2.04)	0.77 (0.59–1.02)*
**Satisfaction with financial situation**						
Completely dissatisfied (1–5)	1	1	1	1	1	1
Moderately satisfied (6–7)	0.39 (0.26–0.59)***	0.28 (0.20–0 .41)***	0.55 (0.36–0 .84)**	0 .37 (0 .30–0 .46)***	0 .39 (0 .25–0.60)***	0.38 (0.33–0.44)***
Completely satisfied (8–10)	0.27 (0.16–0.46)***	0.24 (0.16–0 .38)***	0.59 (0.33–1.09)	0 .30 (0 .24–0 .38)***	0 .20 (0 .10–0 .42)***	0.29 (0.24–0.35)***
**Scales of income**						
Lowest Income (1–4)	1	1	1	1	1	1
Medium Income (5–6)	0.48 (0.33–0 .69)***	0.64 (0.45–0.91)**	0.58 (0.38–0 .89)**	0 .58 (0 .46–0 .72)***	0 .43 (0 .29–0 .63)***	0.55 (0.47–0.63)***
High Income (7–10)	0.47 (0.29–0 .76)***	0.70 (0.46–1.09)	0.31 (0.15–0.63)**	0 .58 (0 .44–0 .76)***	0 .67 (0 .40–1.12)	0.58 (0.48–0.70)***
**Social class**						
Upper class	1	1	1	1	1	1
Upper middle class	0.75 (0.34–1.70)	1.13 (0.35–3.69)	0.34 (0.08–1.37)	2.16 (0 .49–9.43)	0 .56 (0 .23–1.37)	0.75 (0.48–1.19)
Lower middle class	0.61 (0.27–1.38)	0.96 (0.31–3.02)	0.46 (0.12–1.73)	3.57 (0 .83–15.2)	0 .79 (0 .35–1.80)	0.91 (0.59–1.42)
Working class	0.48 (0.21–1.11)	1.13 (0.36–3.56)	0.37 (0.09–1.40)	3.56 (0 .83–15.1)	0 .77 (0 .32–1.81)	0.86 (0.55–1.33)
Lower class	0.66 (0.28–1.57)	0.89 (0.28–2.81)	0.52 (0.14–1.95)	8.17 (1.93–34.6)**	0 .99 (0 .42–2.32)	1.35 (0.87–2.09)
**Country (ref: Nigeria)**						1
Ghana						0.98 (0.80–1.20)
Rwanda						0.56 (0.45–0.70)***
South Africa						1.50 (1.27–1.78)***
Zimbabwe						0.85 (0.70–1.04)

aOR: Adjusted OR.

** p<0.05.

***p<0.0001.

*p<0.10.

**Table 4**exhibits the CI and the percentage contributions of selected socioeconomic characteristics to wealth-based inequalities in poor SRH. In all the study countries, the CI was negative (all p<0.05), implying that poor SRH is excessively concentrated among the poorer socioeconomic strata. The percentage contribution of each of the socio-economic related variable to the poor health CI were different across the five countries. For example, the contribution of participants’ level of education to the poor health CI was highest in Nigeria (23.5%) and lowest in Rwanda (-6% approximately). Regarding satisfaction with financial situation, its contribution to poor health CI was also highest in Nigeria (68%) and lowest in Rwanda (10%). The relative contribution of perceived level of income to poor health CI was highest in Rwanda (63%) and lowest in South Africa (-1.7%). However, the contribution of social class to poor health CI was highest in South Africa (34.8%) and lowest in Nigeria (-8%).

**Table 4:** Relative contribution of selected socioeconomic characteristics to wealth-based inequalities in poor self-rated health.

Variables	Ghana	Nigeria	Rwanda	South Africa	Zimbabwe
	N = 1552	N = 1759	N = 1527	N = 3531	N = 1500
	%	%	%	%	%
Concentration index (95% CI)	-0.13 (-0.16, -0.10)**	-0.19 (-0.21, -0.17)**	-0.38 (-0.40, -0.37)**	-0.18 (-0.20, -0.17)**	-0.19 (-0.22, -0.16)**
Gender	4.20	0.59	-0.08	0.02	0.49
Age, y	13.71	3.66	0.30	6.41	20.96
Marital status	-5.07	4.42	5.42	0.99	11.50
Education	11.59	23.49	-5.95	12.67	9.25
Satisfaction with financial situation	35.70	68.3	10.03	34.68	29.90
Scales of income	24.18	7.46	63.33	-1.68	4.17
Social class	14.74	-8.15	22.89	34.82	22.98
Residual	0.75	0.17	0.35	14.60	0.52

** p<0.05.

Similarly, in **Table 5,** the negative signs of the CI across all the five countries suggest that unhappiness was excessively concentrated among the poorer socioeconomic strata. The contribution of satisfaction with financial situation to unhappiness CI was lowest in Rwanda (19.7%) and highest in Nigeria (65.1%). Further, the percentage contribution of level of income to unhappiness CI was highest in Ghana (64.6%) followed by Rwanda (63.4%) but lowest in South Africa (less than 1%). However, the percentage contribution of social class to the unhappiness CI was highest in South Africa (37.7%) but lowest in Nigeria (-10.5%) and Ghana (-2.8%).

**Table 5:** Relative contribution of selected socioeconomic characteristics to wealth-based inequalities in feeling of unhappiness.

Variables	Ghana N = 1552 %	Nigeria N = 1759 %	Rwanda N = 1527 %	South Africa N = 3531 %	Zimbabwe N = 1500 %
Concentration index (95% CI)	-0.20 (-0.22, -0.18)**	-0.19 (-0.21, -0.17)**	-0.29 (-0.31, -0.27)**	-0.27 (-0.28, -0.27)**	-0.23 (-0.26, -0.21)**
Gender	-0.62	0.08	0.10	0.001	-2.37
Age, y	1.65	8.59	2.32	-0.15	4.92
Marital status	-1.48	-1.49	8.61	0.08	4.72
Education	7.90	7.40	-0.92	4.64	-3.72
Satisfaction with financial situation	30.20	65.10	19.68	36.30	32.05
Scales of income	64.60	38.18	63.39	0.14	45.70
Social class	-2.78	-10.47	5.55	37.69	17.71
Residual	0.40	0.10	1.13	22.40	0.78

** p<0.05.

## Discussion

This paper sought to measure and compare the extent of socioeconomic-related inequalities in SRH and feeling of happiness (subjective wellbeing) in 5 SSA countries (Nigeria, Ghana, South Africa, Rwanda and Zimbabwe) using data from the World Values Survey 2010/2014. To our knowledge, it is one of the first empirical analyses that applied the CI and decomposition analysis to measure the degree of socioeconomic-related inequalities in health and well-being among adult populations in SSA.

Our pooled data analysis showed that people who are moderately or completely satisfied with their financial conditions and those who reported medium or high level of income were less likely to report poor health and unhappiness even after controlling for demographic covariates. By stratifying analysis by country, the association between income levels and health remained consistent for only Rwanda with the rest showing no statistical association. These results are in good agreement with those of previous investigations [1, 14, 16, 30]; which have reported the positive effect of income on health and wellbeing at both individual and regional level.

The statistical association we observed for the aggregated data may perhaps be the result of an increased statistical power. Overall, these results suggest the potential benefits of improved economic conditions to human health and wellbeing. In that regard, authors Matz et al [31] have observed individuals’ spending capacity to positively correlate with higher levels of life satisfaction. This corroborates that prevalence of unhappy status was higher for elderly [32] or low income elderly [33], although as Ye et al. [34] suggested that the traditional economic factors and demographics have low explanatory power over the cross-country differences in subjective well-being. The results further suggest that policies and programs aiming at improving financial conditions of households may end up improving overall health and wellbeing. One may argue from theoretical basis [11] that, in resource constrained settings, wealth may bring prestige to those who earn it and eventually enhance their social standing. Additionally, although money may not buy happiness [35], however it makes healthcare more accessible especially in regions like SSA with massive income inequalities [19] and scarce healthcare resources.

In contrast to reports from previous studies, our findings indicated weak and inconsistent associations between educational attainment and health and happiness across the five countries. This result is quite unexpected and suggests that the educated are not necessarily the healthiest and happiest at country level. This result appears to contradict those of previous studies which found educational gradient in SRH and wellbeing [1, 13, 14]. For instance, in their study of 57 low, middle and HICs, Hosseinpoor et al. (2006) found a descending gradient in the prevalence of poor health as individuals move from low to high educated group [22]. Furthermore, Bann et al. [36] in a British birth cohort study showed that higher education has a beneficial and independent contribution to SRH, lower BMI and many other health-related behaviours. However, as for Lynch, von Hippel [37], the effect of education on SRH differ across age categories and would have greater effect on health at older age. Given our findings, one may argue that education alone may provide little benefit to health and wellbeing if some economic drivers such income and financial conditions remained unsatisfactory. Considering health as basic human right, it is obvious that people need improved level of income and improved financial conditions to be able to purchase healthcare they need for themselves and their dependents. In this study, social class as a socioeconomic marker was associated with feeling of unhappiness in only one of the 5 countries (i.e. South Africa). Thus, being a South African and belonging to a lower social class is associated with eight folds increase in the risk of being unhappy. We argue that the country background could be one potential explanation to this finding. Thus, taking South Africa as one typical example, the data suggests that despite the country’s economic standing, the marks of long history of racial segregation remain an issue.

The use of CI in this paper has offered interesting clues regarding wealth-based inequalities in poor SRH and feeling of unhappiness across the study countries. The negative values of CI observed for all the five countries indicate that poor health and unhappiness are often reported by people of low socioeconomic status. In the present paper, by further decomposing CI, the degree to which each socioeconomic variable contributes to inequalities in SRH and happiness became apparent. Thus, we noticed that among all the covariates, socioeconomic variables relatively contribute more to inequalities in health and happiness, although the magnitudes of their contribution differ by country. In majority of the countries, self-reported satisfaction with financial situation and perceived level of household income were key contributors to poor health and unhappiness concentration indices.

These results are in support of findings reported from Tehran (Iran) by Saharnaz et al [30], who also observed high concentration of sub-optimal SRH among participant of low socioeconomic standing. The results are also in consensus with the notion that health and wellbeing follow a social gradient [9], the socioeconomically better-offs often tend to enjoy better health [1]. Overall, findings of this present investigation have clearly demonstrated the potential significance of socioeconomic-related factors for the promotion of health and wellbeing in the study countries.

## Conclusion

This study is not without limitations. First, our measure of SRH relies on self-reported information; as question on SRH was based on previous timeline thus we cannot rule out the occurrence of recall bias. Second, our findings relied on cross-sectional information, thereby we may not capture the induction time between the onset of overall and wealth based inequalities and their impact on SRH and happiness. In the same way, the causal direction between some predictors such as: marital status, respondent’s education, satisfaction with financial situation, scales of income or social class and SRH cannot be established. Despite those drawbacks, the present study has some strength.

First, our findings rely on most recent nationwide representative samples of respondents. Second, our study compared five countries from SSA region showing the relative contribution to wealth-related and overall inequalities which were based on regression methods as it pertains to SRH and happiness, to our knowledge this is one of the first studies in the context of SSA. Gearing towards policy implications in improving health equality, the commission of social determinants of health indicated three optimal actions among which is “to fight against the unfair distribution of resources” [9]. Therefore, knowledge about the self-rating of health may serve as a proxy in understanding the distribution of health care services and other necessary resources relevant for population health in resident countries.
